# Complete genome reconstruction of the global and European regional dispersal history of the lumpy skin disease virus

**DOI:** 10.1128/jvi.01394-23

**Published:** 2023-10-31

**Authors:** Steven Van Borm, Simon Dellicour, Darren P. Martin, Philippe Lemey, Eirini I. Agianniotaki, Eleni D. Chondrokouki, Dejan Vidanovic, Nikola Vaskovic, Tamaš Petroviċ, Sava Laziċ, Xhelil Koleci, Ani Vodica, Igor Djadjovski, Kiril Krstevski, Frank Vandenbussche, Andy Haegeman, Kris De Clercq, Elisabeth Mathijs

**Affiliations:** 1 Scientific Directorate Animal Infectious Diseases, Sciensano, Brussels, Belgium; 2 Spatial Epidemiology Lab (SpELL), Université Libre de Bruxelles, Brussels, Belgium; 3 Laboratory for Clinical and Epidemiological Virology, Department of Microbiology, Immunology and Transplantation, Rega Institute, KU Leuven, Leuven, Belgium; 4 Institute of Infectious Diseases and Molecular Medicine, University of Cape Town, Cape Town, South Africa; 5 National Reference Laboratory for Capripoxviruses, Department of Molecular Diagnostics, FMD, Virological, Rickettsial and Exotic Diseases, Directorate of Athens Veterinary Center, Ministry of Rural Development and Food, Athens, Greece; 6 Department for laboratory diagnostics, Veterinary Specialized Institute, Kraljevo, Serbia; 7 Department for Virology, Scientific Veterinary Institute, Novi Sad, Serbia; 8 Faculty of Veterinary Medicine, The Agricultural University of Tirana, Tirana, Albania; 9 Animal Health Department, Food Safety and Veterinary Institute, Tirana, Albania; 10 Faculty of Veterinary Medicine, Ss. Cyril and Methodius University in Skopje, Skopje, Macedonia; Cornell University Baker Institute for Animal Health, Ithaca, New York, USA

**Keywords:** phylogeography, molecular epidemiology, *Capripoxvirus*, lumpy skin disease virus, recombination

## Abstract

Lumpy skin disease virus (LSDV) causes a disease of economic importance affecting cattle. Its global epidemiology is complex due to the combination of vector-borne and anthropogenic spread, the circulation of vaccine-like recombinants, and the use of vaccines. The slow molecular evolution of its DNA genome limits the utility of genetic variation for accurate tracing based on evolutionary analyses, but this limitation has not yet been formally assessed. Furthermore, until present, whole genome sequencing in affected areas has remained patchy. This study combines the first fine-grained sampling of LSDV whole genomes from a time-constrained (2015–2017) southeastern European (SEE) LSDV outbreak, which we analyze along with curated public genomes to investigate the global and regional viral dispersal dynamics. First, haplotype networks visualizing the limited genetic variability associated with the SEE LSDV outbreak show intense intermixing between countries. We also assess at which spatial scale a correlation between genetic and geographic distances can be detected for LSDV. On a global scale, we show the importance of accounting for recombination events that can impact phylogenetic and phylogeographic reconstructions. Following the assessment of the temporal signal in the recombination-free alignment, our time-scaled continuous phylogeographic analysis of Kenya-like and recent wild-type viruses confirms the origin and global dissemination history of LSDV. Our analyses highlight the importance of careful selection and application of phylodynamic approaches to DNA viruses, as well as the importance of whole genome sampling in endemic and outbreak areas to improve our understanding of the evolution, epidemiology, and transmission dynamics of DNA viruses.

**IMPORTANCE:**

Lumpy skin disease virus (LSDV) has a complex epidemiology involving multiple strains, recombination, and vaccination. Its DNA genome provides limited genetic variation to trace outbreaks in space and time. Sequencing of LSDV whole genomes has also been patchy at global and regional scales. Here, we provide the first fine-grained whole genome sequence sampling of a constrained LSDV outbreak (southeastern Europe, 2015–2017), which we analyze along with global publicly available genomes. We formally evaluate the past occurrence of recombination events as well as the temporal signal that is required for calibrating molecular clock models and subsequently conduct a time-calibrated spatially explicit phylogeographic reconstruction. Our study further illustrates the importance of accounting for recombination events before reconstructing global and regional dynamics of DNA viruses. More LSDV whole genomes from endemic areas are needed to obtain a comprehensive understanding of global LSDV dispersal dynamics.

## INTRODUCTION

Lumpy skin disease virus [LSDV; Poxviridae, *Capripoxvirus* genus (1)] is the causative agent of an important viral disease of cattle and water buffalo, lumpy skin disease (LSD). LSDV has a linear double-stranded DNA genome approximately 151 kb in length (2). The *Capripoxvirus* genus (CaPV) hosts two additional members: the goatpox virus (GTPV) and the sheeppox virus (SPPV), whose genomes show over 96% nucleotide identity to LSDV (3). LSD causes severe economic losses to the cattle sector due to morbidity, production losses, and control measures. As a consequence, the disease has been listed as notifiable by the World Organization for Animal Health (4). Arthropods provide the main route of LSDV transmission [reviewed in references (5, 6)]. The disease was first reported in 1929 in northern Rhodesia (current Zambia), reached South Africa in 1944 (7), and became an endemic disease in many countries of sub-Saharan Africa during the last century [reviewed in references (5, 8)]. During the last decades, the disease moved north and eastward to become endemic in the Middle East and Turkey (8, 9). The first LSDV incursion on the European continent was recorded in Greece in August 2015 near the Turkish border (10), followed by spread across the Balkan peninsula of continental Europe, including Greece, Bulgaria, North Macedonia, Albania, Kosovo, Serbia, and Montenegro (5, 8, 11). Simultaneously, outbreaks were reported in the Caucasus region and Kazakhstan. In southeastern Europe, after a decline in the reported outbreaks in 2017, no outbreaks were reported in 2018 and thereafter, confirming the effectiveness of a high-coverage (>80%) mass vaccination campaign in affected and neighboring countries with a live attenuated LSD vaccine based on the Neethling strain (9, 12).

Both homologous (i.e., LSDV-based) and heterologous (i.e., GTPV- or SPPV-based) live attenuated vaccines are used in LSD control efforts worldwide (13). Homologous vaccines are based on historical LSDV isolates from South Africa (Neethling strain) and Kenya [“Kenyan sheep and goat pox*”* — KSGP-based strain, identified as LSDV using genomic approaches (14, 15)] following classical attenuation approaches using serial passaging in cell cultures and the chorioallantoic membrane of embryonated chicken eggs (16). Neethling-based vaccines provide good protection against virulent LSDV strains but can cause mild adverse reactions in cattle referred to as “Neethling response” (local reaction at the vaccination site and, more rarely, generalized skin lesions) (13, 17, 18). KSGP-based vaccines have been successfully used as heterologous vaccines against SPPV and GTPV in small ruminants but can cause clinical signs in vaccinated cattle (13, 17).

Two to three years after the occurrence of LSDV in the Caucasus region and central Asia in 2014–2015, vaccine-like recombinant strains were detected in Russia (19, 20). Genomic analyses differentiated these from the wild-type strains typical of the LSDV outbreaks in Europe and the Middle East (19). The clinical features of the disease caused by these vaccine-like recombinant strains resembled the signs of the clinical disease in outbreaks caused by the wild-type LSDV strains. Molecular diagnostic assays for the differentiation of the Neethling LSDV vaccine and the wild-type LSDV strains had to be updated due to the emergence of these recombinant vaccine strains (21). The spread of these vaccine-like recombinants was hypothesized to be linked to the use of live Neethling strain vaccine in Kazakhstan (22) and either illegal movement of vaccinated animals or unauthorized vaccination in Russia (23). Detailed genomic analyses of vaccine batches used in Kazakhstan (22) suggest recombination events occurred during vaccine production due to the presence of multiple CaPV strains in vaccine seeds rather than after co-infection in cattle in the field (24). Although no evidence exists for the recombination of LSDV in multiple infected cattle in the field, up to five different vaccine-like recombinants were detected in cattle in Russia and Kazakhstan between 2017 and 2020 (24, 25), one of which [R4 (24)] spread to large parts of Asia (26, 27). Surprisingly, even though the importance of recombination-induced bias in phylogenetic analyses has been repeatedly stressed (28, 29), whole genome-based LSDV phylogenetic analyses have, until present, not been controlled for this effect [e.g., references (25, 30, 31)]. Further complicating the epidemiological picture, LSDV genomes characterized in India and Bangladesh suggest a direct link with East African wild-type LSDV strains rather than vaccine-like recombinants circulating in large parts of Asia (32, 33).

The slow molecular evolution of Orthopoxviruses [between 6.7 × 10^−6^ and 1.1 × 10^−5^ substitutions/site/year as estimated for Variola virus (34)] provides a challenge for exploring the evolutionary history of those viruses using phylodynamic approaches. Specifically for LSDV, one study compared the full genome of one historical and one contemporary South African LSDV isolate, estimating the substitution rate as 7.4 × 10^−6^ substitutions/site/year (35). Such a low substitution rate could restrict the accumulation of enough genetic variability to accurately trace the dispersal history of lineages using phylogeographic analyses; a potential limitation that has never been formally assessed in the case of LSDV. Due to the low substitution rates of CaPV, generally, there will be only sufficient genetic variability within small genome regions (such as individual genes) to differentiate between the most divergent viral lineages. Therefore, such studies in southeastern Europe, differentiate between the Neethling vaccine LSDV strains and the wild-type LSDV strains. However, partial genomic sequences cannot be reliably used to differentiate the southeastern European LSDV lineages from one another [e.g., reference (36)].

The global epidemiological context of LSDV is complex. Virus transmission is mediated by a naturally broad range of blood-sucking arthropod vectors and the transport of cattle to naive regions. In addition, vaccination is applied, and both natural viral lineages, vaccine-derived lineages, and recombinant lineages (including recombinants of different vaccine strains) circulate. It would, therefore, be very useful from the perspective of formulating and evaluating control strategies if it was possible to use molecular epidemiology-based approaches to track the global and local dissemination of LSDV lineages using genome sequence data. In this context and given the generally low degrees of genetic diversity evident within the circulating LSDV lineages, whole genome sequence-based analyses would be needed to maximize the potential utility of such molecular epidemiological analyses.

Previously available genomic data sets for LSDV often lacked extensive sampling of full genome sequences or failed to take recombination events into account in their analyses (30, 31). Adding to this patchy global sampling of full LSDV genomes, our study generated the first fine-grained full genome sampling and sequencing of the southeastern Europe 2015–2017 incursion of LSDV to maximize the amount of genetic information that can be gained from this slowly evolving DNA virus. Specifically, we aimed to establish to what extent LSDV full genomes can unravel the dispersal history and recombination dynamics of the virus at global and more regional spatial scales, taking into account the bias that can be introduced when not accounting for recombination events.

## MATERIALS AND METHODS

### Sampling and metadata curation

Sample repositories from LSDV-affected countries were investigated for LSDV-positive samples that had associated vaccination status, sample location, and sampling date metadata. Samples were verified as being LSDV positive (real-time qPCR assays according to the standard operating procedures of contributing national laboratories, details available on request) prior to shipping to Sciensano’s BSL3 facility for centralized LSDV whole genome sequencing (WGS). Legal, ethical, and biosafety requirements were met according to national and European legislation prior to sending the samples. Upon arrival, DNA was extracted and verified using a pan-CaPV real-time PCR assay targeting the conserved D5R region (nomenclature according to VACV strain Copenhagen M35027.1 ortholog) (37), and only samples with sufficient LSDV concentrations (associated Cq scores < 30) were used for sequencing; a stringency that resulted in the rejection of screened samples from Montenegro and Azerbaijan. Between two and six skin biopsy samples per country (Albania *n* = 6, August–December 2016; Greece *n* = 5, November 2015–November 2016; North Macedonia *n* = 2, April–May 2016; Serbia *n* = 5, June–September 2016) were selected for additional whole genome sequencing to maximize the spatiotemporal sampling coverage (see Table S1 for the list of samples, associated metadata, and GenBank accession numbers).

### DNA extraction and targeted CaPV whole genome sequencing

DNA extraction, LSDV genome amplification, massive parallel sequencing, and genome assembly were performed as previously described (38). Briefly, DNA was extracted from skin sample homogenates using a Puregene extraction kit (Qiagen), followed by PCR amplification of the genome by long-range PCR (amplicons of approximately 7,500 bp). Amplicons were equimolarly pooled per half genome and sequenced using a Nextera XT DNA Library Preparation Kit (Illumina) and a MiSeq Reagent Kit version 3, 2 × 300 bp (Illumina) as previously described (38). The sequencing run was performed at the Neuromics Support Facility–VIB Genomics Core (UAntwerp, Belgium). Half genomes were *de novo* assembled and combined into complete genomes as described in reference (38). Discrepancies between the full-length consensus sequences and the genome sequence of LSDV isolate Evros/GR/2015 (KY829023.3, genome of the first incursion of LSDV in Europe in 2015) were confirmed or refuted by dideoxy chain terminator sequencing (Sanger sequencing) as previously described (38). Sanger sequencing was performed by the transversal activities in the applied genomics service of Sciensano on an Applied Biosystems 3130 Genetic Analyzer Sequencer using the BigDye Terminator v3.1 Cycle Sequencing Kit (Thermo Fisher Scientific) according to the manufacturer’s instructions. Annotation and amino-acid gene prediction of finished genomes were performed using GATU software (39) relative to the LSDV field isolate Evros/GR/2015 (KY829023.3) for wild-type viruses and Neethling-Herbivac (KX764644.1) for Neethling-like viruses. In total, 18 complete coding sequences were generated and submitted to GenBank (see Table S1 for accession numbers).

In addition to this study’s sequencing effort, all near-complete LSDV genomes with a minimum length of 150,000 bp were extracted from the NCBI nucleotide database (extraction date: 10 March 2023). Sequences with large gaps (>500 nt) were removed to maximize the number of available variable positions in the alignment. The final global LSDV alignment was made of 72 genomes, including 21 genomes from Europe (Table S1). Geographical coordinates were either provided by the sample or genome submitter or retrieved from Google Maps (https://www.google.com/maps/; accessed on 15 March 2023) considering the center of the most precise administrative unit provided (see below regarding the inclusion of sampling uncertainty for the phylogeographic reconstruction). Sampling time was provided by the sample submitter or taken from the public sequence record metadata, with variable precision ranging from the exact sampling date (for all European genomes sequenced in the present study) down to the year. LSDV genomes were aligned using MAFFT v7.310 (40), and the resulting alignment was subsequently trimmed to remove extremities with missing data, reaching a final alignment length of 148,398 nucleotides.

### Recombination analyses

We applied the Φ-test (41) implemented in the program SplitsTree (42) to assess the presence of a recombination signal within our data set. This test is based on the computation of a pairwise homoplasy index that is a measure of the similarity between closely linked sites, and for which the level of significance is assessed by permuting nucleotide sites. We further inferred the position of recombination breakpoints by using the set of methods available in the program RDP4 (43) as well as the GARD method (44) implemented in the program HyPhy (45)

### Phylogenetic analyses

We inferred maximum likelihood (ML) phylogenetic trees using the program IQ-TREE 1.6.12 (46). The ML tree was inferred under a general time-reversible (GTR) model of nucleotide substitution with empirical base frequencies and a four-category FreeRate model of site heterogeneity, which was selected as the best-fitting model using IQ-TREE’s ModelTest functionality and 100 bootstrap replicates to assess branch support. We also constructed a haplotype network, an alternative to phylogenetic trees, using the median-joining method (47) implemented in the program Network 5 (available at http://www.fluxus-engineering.com) and plotted with the R function “networkGraph” available with the toolbox SPADS 1.0 (48). The temporal signal associated with the two wild-type clades was assessed by performing the root-to-tip regression analysis implemented in the program TempEst (49) and based on the corresponding ML tree inferred by IQ-TREE, which led to a coefficient of determination (*R* ^2^) of 0.28.

### Population genetic analyses

We performed two different analyses to explore the genetic differentiation and population structure inherent to the two “wild-type” clades, i.e., excluding vaccine-like recombinants and Neethling-like sequences (see the Results section): (i) an analysis of the isolation-by-distance (IBD) pattern (50) and (ii) spatial analysis of molecular variance (SAMOVA), the latter being a clustering algorithm based on the analysis of DNA sequences (51). For the IBD analyses, we performed a Mantel test (52) between an inter-individual distance based on pairwise nucleotide differences between DNA sequences (*IID*2) and the log-transformed great-circle geographic distance between sampling points. Mantel tests were based on 1,000 permutations and performed on different subsets of data (Table 1): when considering (i) the Kenya-like and recent wild-type viruses, (ii) only the recent wild-type clade, (iii) only the recent wild-type clade but when excluding African, Kazakhstan, and Russian samples, (iv) when only considering the European sequences (excluding Turkey), and (v) when only considering the R4 clade of vaccine-like recombinants. The SAMOVA method aims to assign sampling locations to *K* groups based on genetic similarity and geographic vicinity, the most likely structure corresponding to the partition maximizing the among-group differentiation as measured by the Φ_CT_ statistic (53). We performed 100 independent runs of 10,000 simulated annealing steps for each *K* value varying from 2 to 15. The computation of the pairwise inter-individual distances *IID*2 as well as the SAMOVA was performed with the program SPADS 1.0 (48).

**TABLE 1:** Investigation of the isolation-by-distance pattern^*a*^

Subset of genomic sequences	*r*_*S*_ (p-value)
Kenya-like and recent wild-type viruses (clade 1.2)	0.648 (0.001)
Only recent wild-type viruses (clade 1.2b)	0.762 (0.001)
Only recent wild-type viruses (clade 1.2b) but when excluding African, Kazakhstan, and Russian samples	0.515 (0.002)
Only considering the European sequences of clade 1.2b (minus Turkey)	0.100 (0.239)
R4 recombinants clade	0.089 (0.270)

^*a*^ We here report the Spearman correlation coefficient (*r*_*S*_) between the inter-individual genetic distance and the log-transformed geographical distance, along with the associated *p*-values obtained from the corresponding Mantel test.

### Phylogeographic analyses

Despite the moderately low temporal signal assessed by the root-to-tips regression analysis, we were able to calibrate a molecular clock model in a continuous phylogeographic analysis performed in the Bayesian framework of the software package BEAST 1.10 (54) coupled with the BEAGLE 3 library (55) to improve computational performance. Specifically, we used the relaxed random walk diffusion model (56 – 58) implemented in BEAST to infer the dispersal history of wild-type LSDV lineages. The among-branch heterogeneity in diffusion velocity was modeled with a gamma distribution, branch-specific evolutionary rates were modeled according to a relaxed molecular clock with an underlying lognormal distribution, the nucleotide substitution process was modeled according to a GTR + Γ parameterization, and we also specified a flexible skygrid model as the tree prior (59). The Markov chain Monte Carlo algorithm was run for 10^9^ generations while sampling every 10^5^ generations. Convergence and mixing properties were assessed using the program Tracer 1.7 (60), and that effective sampling size values associated with continuous parameters were all >200. After having discarded 10% of sampled posterior trees as burn-in, we obtained and annotated the maximum clade credibility (MCC) tree using the program TreeAnnotator 1.10.4 (54). We used functions available in the R package “seraphim” (61, 62) to extract spatiotemporal information embedded within posterior trees and visualize the continuous phylogeographic reconstructions.

Because of the lack of precision for the sampling location of a number of genomic sequences retrieved from GenBank, we used a uniform sampling prior approach to define a potential area of origin for these sequences (63, 64). This was the case for three genomic sequences from Kenya (AF325528.1, KX683219.1, and MN072619.1), one sequence from Namibia (MT007950.1), one sequence from India (OP297402.1), and one sequence from Israel (KX894508.1), for which we integrated sampling coordinates across the entire country; as well as for one sequence from the KwaZulu Natal province in South Africa (MW656253.1), two sequences from the Mymensingh division in Bangladesh (OP688128.1, OP688129.1), one sequence from the Dagestan autonomous republic in Russia (MH893760.2), one sequence from the Atyrau region in Kazakhstan (MN642592.1), one sequence from the Tokat province in Turkey (MN995838.1), and one sequence from the Yambol province in Bulgaria (MT643825.1), for which we integrated the sampling coordinates from the corresponding administrative polygon retrieved from the Database of Global Administrative Areas (GADM, https://gadm.org/).

## RESULTS

### Description of the generated genomic data set

**Fig 1:**
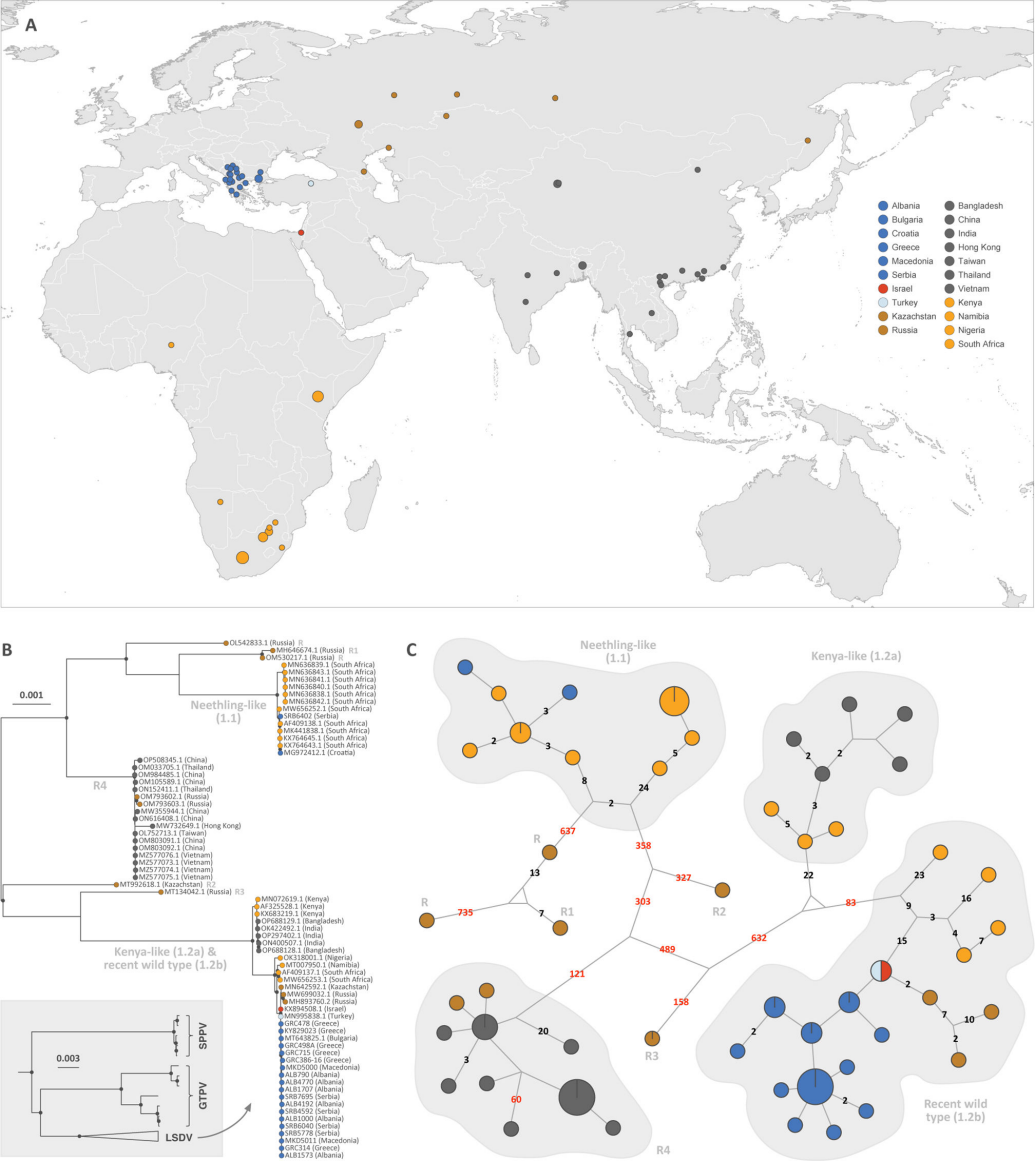
Sampling map and genetic variability of LSVD genomes. (**A**) Sampling map. (**B**) ML phylogenetic tree based on the entire alignment for which the internal nodes are highlighted only when the associated bootstraps support is higher than 70% (see Fig. 2 for a comparison with the ML tree based on the recombination-free alignment). (C) Haplotype network based on the entire alignment. In the network, each haplotype corresponds to a unique sequence represented by a circle, the size of which is proportional to its overall sampling frequency, and the genetic relatedness between haplotypes is represented by line segments. If more than one mutational change separates two haplotypes, a number indicates the number of mutations (see Fig. 2 for the haplotype network focused on the southeastern European clade).

This study resulted in 18 high-quality near-complete LSDV genome assemblies from southeastern Europe (2016–2017). Between two and six LSDV genomes were completed from North Macedonia, Greece, Albania, and Serbia. After confirmation with Sanger sequencing and annotation of the genomes, the sequences were submitted in GenBank under accession numbers OR134832, OR134833, OR134834, OR134835, OR134836, OR134837, OR134838, OR134839, OR134840, OR134841, OR134842, OR134843, OR134844, OR134845, OR134846, OR134847, OR134848, and OR134849 (see Table S1 for details and associated metadata). All sequences were characterized by a 145,885 bp central coding region, flanked by two inverted terminal repeats of at least 2,164  bp, and contain all expected LSDV open reading frames. They shared a high pairwise nucleotide sequence identity ranging between 0.984 and 1.000. The addition of complete and gap-free public LSDV genome sequences resulted in an alignment of 72 complete LSDV genomes including 21 from the southeastern European 2015–2017 epidemic (Fig. 1A; Table S1). Of note, the Greece/Evros/2015 (10), Bulgaria/2016 (65), Israel/2012 (KX894508.1), and four Vietnamese (66) LSDV genomes were previously sequenced by our group and the genome assembly included Sanger sequencing confirmation of variant positions.

**Fig 2:**
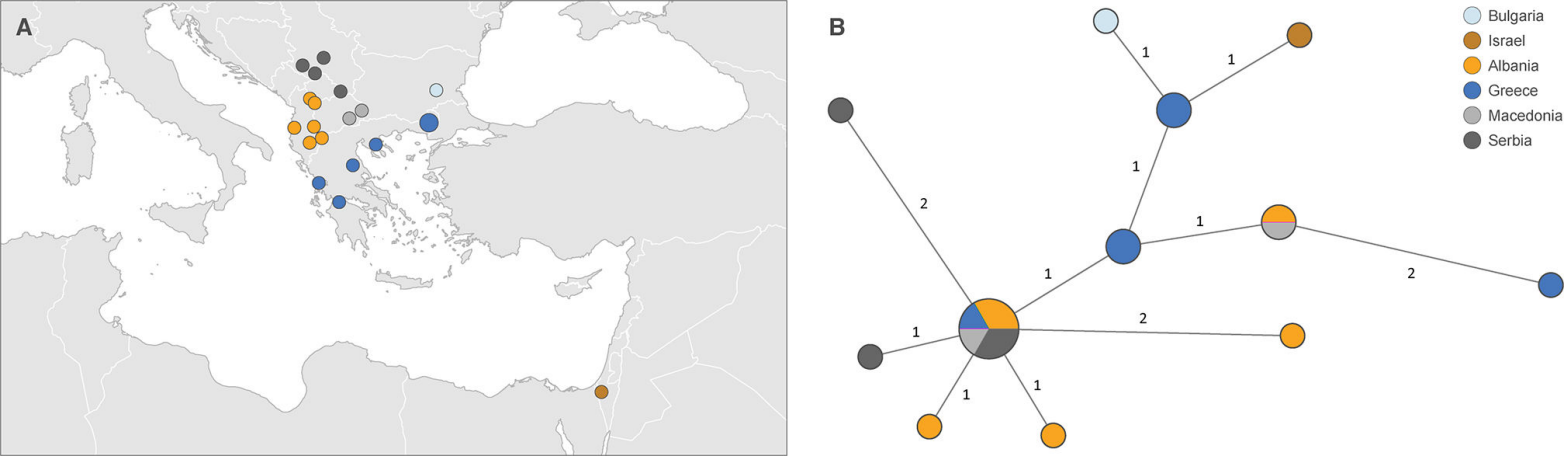
Sampling map and genetic variability of LSDV genomes within the southeastern European outbreak. (**A**) Sampling map. (**B**) Haplotype network of wild-type LSDV dispersal during the southeastern European outbreak. In the network, each haplotype corresponds to a unique sequence represented by a circle, the size of which is proportional to its overall sampling frequency, and the genetic relatedness between haplotypes is represented by line segments. If more than one mutational change separates two haplotypes, a number indicates the number of mutations.

### Visualization of the LSDV evolutionary history

The neutral definition of the term “clade” was used, as any part of a phylogeny including an ancestral lineage and all the descendants of that ancestor. The ML phylogeny (Fig. 1B) and haplotype network (Fig. 1C) are both in agreement with the history of LSDV spread documented in disease reports and partial sequencing data. Neethling strain-like historical and vaccine viruses [clade “Neethling-like,” corresponding to “subgroup 1.1” in the terminology proposed by Biswas and colleagues (31)] are clearly distinguished from Kenya-like and recent wild-type viruses [“subgroup 1.2” according to reference (31)] and recombinant viruses. Two European LSDV genomes within this “Neethling-like” clade represent cases with adverse reactions of Neethling-based live attenuated vaccines and clearly cluster with Neethling vaccines (Fig. 1B and C). This includes a single case from Serbia (SRB6402) sequenced in this study as well as a sequence from Croatia (MG972412.1), both in 2016. In addition, a series of South African LSDV virulent Neethling-like strains from the 1990s (67, 68) clearly cluster in the Neethling-like clade (Fig. 1B). Of note, a series of recently released genomes from historical (1958–1977 (69)) Neethling-like field isolates were not publicly available at the time of our analyses. Interestingly, the haplotype network suggests an early differentiation of these virulent viruses from the Neethling vaccine attenuation history (Fig. 1C).

Within the Kenya-like and recent wild-type viruses clade [corresponding to “subgroup 1.2” in the terminology proposed in reference (31)], a well-supported clade containing viruses from East Africa, India, and Bangladesh (clade 1.2a; Fig. 1B and C) is separated from recent wild-type viruses from southern Africa, central Asia, the Middle East, and Europe (clade 1.2b; Fig. 1B and C). Within the clade 1.2a, the Indian and Bangladeshi LSDV genomes show only a limited number of nucleotide differences to a genome from a commercial East African vaccine derived from a Kenyan 1974 strain (KX683219.1; Fig. 1C). Within clade 1.2b, the ML tree provides limited resolution at the European scale (Fig. 1B). The haplotype network (Fig. 1C) visualizes the differences between sequence variants on a finer geographical scale and highlights the pivotal role of the Middle East in the spread of LSDV from Africa towards both Europe and Central Asia. Within the southeastern European outbreak, LSDV genomes differ by a maximum of five single nucleotide substitutions, without evidence for clustering per country (Fig. 2). Eleven unique haplotype variants are present in the population of 19 wild-type samples from southeastern Europe. A single haplotype is shared by samples from four sampled countries (Albania, Greece, North Macedonia, and Serbia) and represents 6 of the 19 sampled wild-type genomes (31.6%). A second haplotype is shared between Albania and North Macedonia. In addition, all countries except North Macedonia (where only two genomes were sequenced) show multiple unique haplotype variants within the country (Fig. 2).

**Fig 3:**
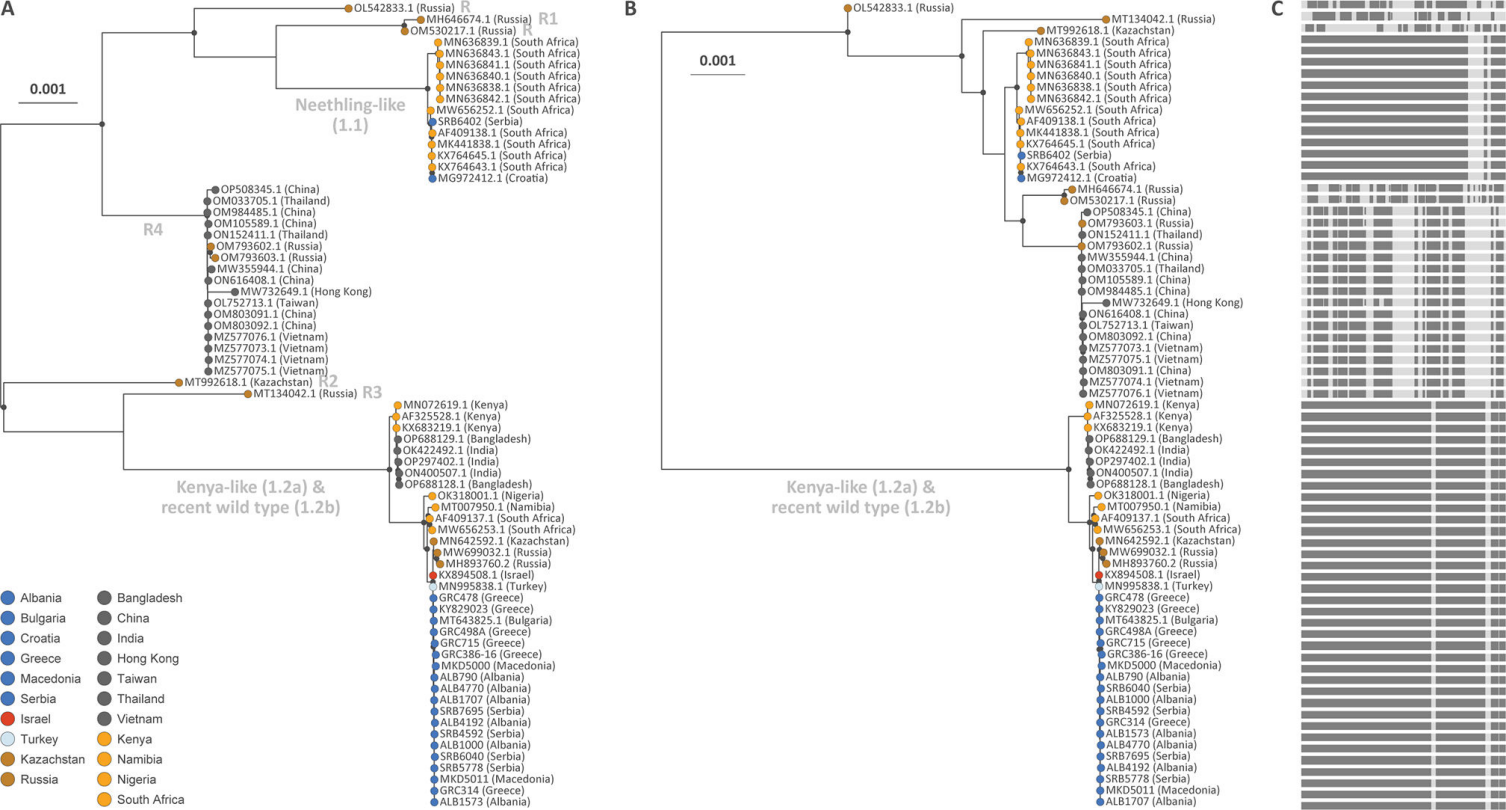
Comparison between the ML tree based on the overall alignment (**A**) and the one based on the recombination-free (**B**) alignment. In both trees, we only highlight the internal nodes for which the associated bootstrap support is higher than 70%. (**C**) Next to the ML tree based on the recombination-free alignment, we also display a schematic whole genome view of the recombination-free genomic regions that were retained in the alignment (darker gray bands) and the recombination signals (lighter gray bands) that were removed from the alignment.

We confirm the circulation of multiple vaccine-like recombinants (but see below for their phylogenetic analysis after correction for recombination events) and refer to the terminology proposed by Vandenbussche and colleagues (24). A single vaccine-like recombinant clade R4 groups the genomes representing the spread of this recombinant virus to large parts of Asia (Fig. 1B and C). The unexpected long distance (Fig. 1B), even after removing recombination signals (Fig. 3), and number of single nucleotide polymorphisms (SNPs) (Fig. 1C) to MW732649.1 from Hong Kong (60 SNPs) and OP508345.1 from Xinjiang (20 SNPs) suggest potential issues with the assembly of those genomes.

### Detection of recombination events in the LSDV global evolutionary history

We observed a significant signal for the occurrence of past recombination events (Φ-test, *P* < 0.001), and up to 78 past recombination events were identified by RDP4 (see Fig. 3 for a graphical overview of the recombinant regions). To avoid bias of recombination events in the phylogenetic reconstructions, we repeated the ML-based reconstruction after removing recombination signals from the alignment using RDP4 (Fig. 3). In the absence of recombination signals (Fig. 3B), all previously identified recombinant genomes significantly cluster with the Neethling-like clade. This supports the conclusion that the recombinants originated from a Neethling-like virus. The visualization of removed recombination signals by RDP4 (Fig. 3C) confirms the presence of five different recombinants. We use the “R1–R4” terminology proposed by Vandenbussche et al. (24), while R5 is represented by the vaccine-like recombinant described from Tyumen, Russia in 2019 (25). R4 recombinants, spreading to large parts of Asia after their emergence, stand out as a monophyletic group but their close relationship with the Neethling-like clade as well as with the other recombinants OM530217 and MH646674 (R1) from Russia are now emphasized. The phylogenetic clustering of the reemergence of R1 at the same geographical location (Saratov, Russia, 2017 and 2019) (70) remains unaltered after correction for recombination signals.

### Genetic differentiation and population genetic structure at different geographical scales

We investigated the genetic differentiation of LSDV at different geographical scales and identified a significant isolation-by-distance signal identified within the wild-type clades, but this signal is no longer significant when only considering the European sampling, meaning that at the European scale, we do not find any supported association between the genetic differentiation and the geographic distance (Table 1). Similarly, when only considering the spread of the R4 recombinants in Asia, no isolation-by-distance is evident. We further performed a SAMOVA to investigate the population genetic structure of wild-type LSDV samples. With a Φ_CT_ statistic constantly increasing with the number of considered *K* clusters to delineate, this analysis fails to identify a clear clustering among sampling locations of wild-type genomic sequences. This indicates a lack of detection of a clear population genetic structure among the samples belonging to that clade, rather than highlighting a continuum of genetic variation across the study area.

### Phylogeographic reconstruction of the dispersal history of LSDV lineages

**Fig 4:**
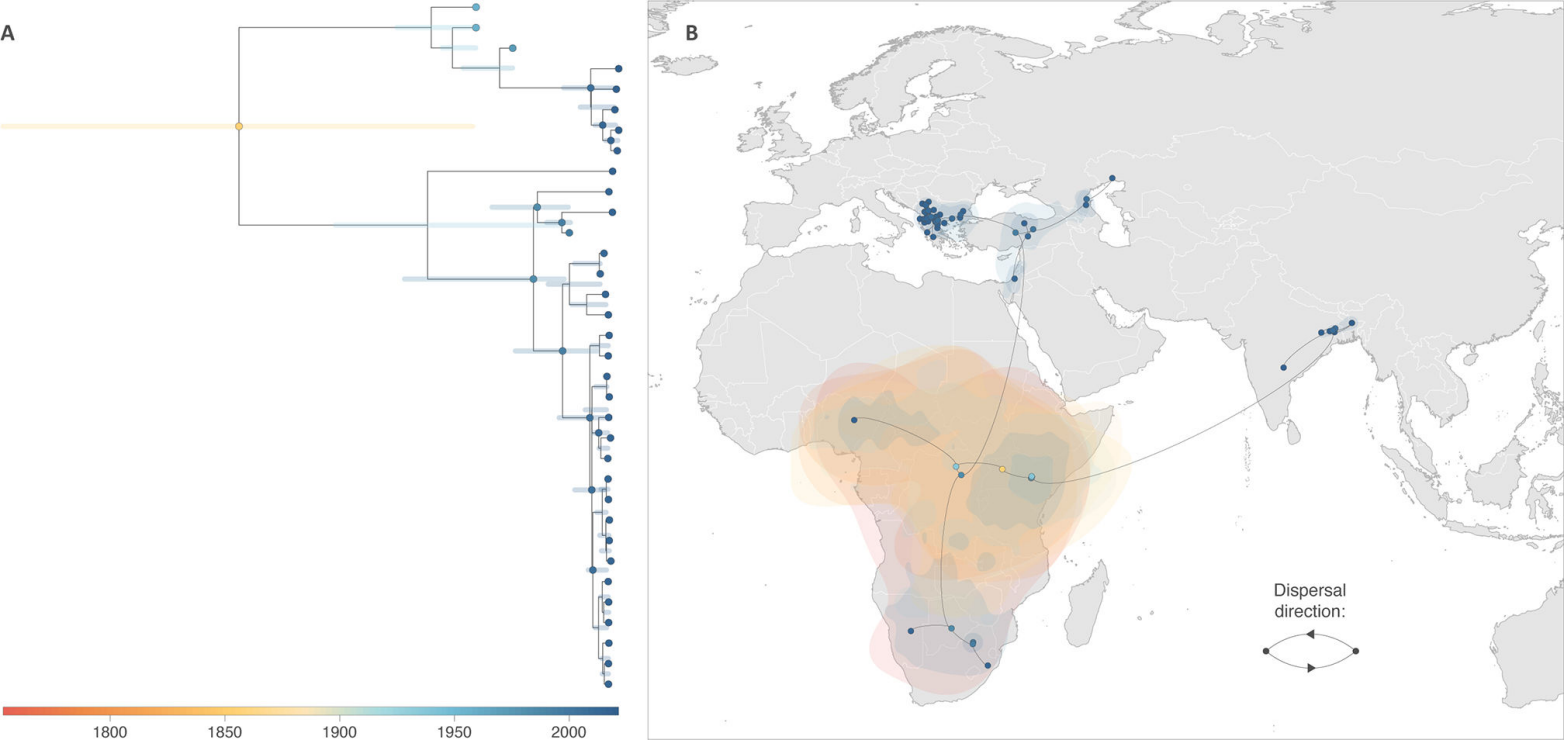
Continuous phylogeographic reconstruction of the wild-type LSDV clades. (A) Time-scaled MCC tree obtained from the continuous phylogeographic inference on which we only highlighted the internal nodes associated with a posterior probability > 0.95. (B) Reconstruction of the dispersal history of LSDV lineages within the wild-type clade. Here, we map the MCC tree and 80% HPD regions reflecting the uncertainty related to the Bayesian phylogeographic inference. MCC tree and 80% HPD regions are based on 900 trees sampled from the posterior distribution of trees and are colored according to their time of occurrence.

The time-scaled phylogenetic inference associated with our continuous phylogeographic reconstruction allows estimating for the clade 1.2 (Kenya-like and recent wild-type viruses) a root age around 1888 [95% highest posterior density, HPD = (1744, 1958); Fig. 4A] and a substitution rate equals to 6.08 × 10^−5^ substitutions/site/year [95% HPD = (4.07 × 10^−7^, 3.38 × 10^−4^)]. The continuous phylogeographic reconstruction of the dispersal history of wild-type LSDV lineages (Fig. 4B) did not highlight multiple long-distance dispersal events among the infected continents, which further illustrates the isolation-by-distance signal estimated across a large spatial scale. While this analysis infers the location of the most ancestral nodes connecting African, Asian, and European clades in Africa, the statistical uncertainty associated with the inferred location is relatively high, preventing to conclude on a precise geographic origin.

## DISCUSSION

The considerable genetic variability of RNA virus populations allows for a detailed phylogeographic tracing within outbreaks [e.g., references (71 – 73)], but the limited genetic variability within DNA virus populations constrains the resolution at which population genetic and phylodynamic investigations can be performed even when based on the analysis of whole genome sequences. To our knowledge, this study presents the first fine-grained sampling of full genomes collected from a regional LSDV epidemic, which results in 21 high-quality complete genomes for an outbreak spanning less than 3 years and an outbreak area as small as the Balkan peninsula of continental Europe. We combined these genomes with a global context of publicly available gap-free complete LSDV genomes.

Previous analyses of LSDV whole genome data sets did not formally evaluate the correlation between geographical and genetic distances [e.g., reference (30)]. Here, we found that although this was the case on a global and inter-regional scales, no association existed between genetic and geographic distances at the regional (southeastern Europe) scale. Different factors may contribute to this lack of geographical signal at the southeastern European scale compared to larger inter-regional scales. These include the slow evolutionary rate of CaPV and the limited geographical area and timeframe of the outbreak due to the successful regional eradication of the disease using mass vaccination with homologous live attenuated vaccines. In addition, intense interconnectivity between affected countries due to a combination of vector-borne and anthropogenic transmission likely resulted in a highly intermixed virus population as also indicated by the lack of clustering in the SAMOVA. Sampling even more densely for whole genomes in this limited outbreak would likely not have resulted in a significant isolation-by-distance signal at a regional scale. As a result, even whole genome sequences of LSDV did not allow tracing in time or space at this regional scale. Of note, this lack of regional isolation-by-distance is specific to the particular epidemic studied in the present study, which took place in a very short timeframe (3 years) and across a relatively small geographical area (maximum great-circle distance between sampling locations: 733 km). Similarly, we show that within the currently available public whole genome data of clade R4 vaccine-like recombinants spreading through East Asia (China, Taiwan, Vietnam, and Thailand), there is no support for isolation-by-distance. In areas of endemic circulation or extended epidemic spread of CaPVs, sufficient genetic variation may result in detailed phylogeographical analyses on a smaller geographical scale.

In the context of limited genetic variation, haplotype networks allow a useful visualization of the genetic differences between sampled genomes in a regional outbreak. These approaches have been recently used for understanding the spatial and temporal relationships between mpox virus (previously monkeypox virus) (MPXV) isolates (74, 75). LSDV is well-adapted to the cattle host, in contrast to the continuing adaptation of the MPXV in the human host potentially affecting the genetic variation (76) visualized in haplotype networks. For LSDV, the network visualization of sequence polymorphisms confirms the presence of multiple unique sequence variants within European countries in such a short timeframe and limited geographical area. However, it also allows visualizing the lack of isolation-by-distance on this smaller geographical scale as there is no clustering per country. On a global scale, genetic distance-based as well as haplotype-based methods offer a meaningful visualization of LSDV dissemination and evolution. Our reconstruction is largely in line with previous reconstructions of global LSDV epidemiology based on epidemiological or (partial) genomic information [reviewed in references (5, 8)]. Our analyses confirm previous phylogenetic findings for Neethling strain LSDV vaccines with a linked cluster representing the circulation of virulent but phylogenetically vaccine-like LSDV lineages from outbreaks in South Africa during the 1990s as previously described (67). A recent study by van Schalkwyk and colleagues showed that the complete genomes of historical (1958–1977) South African virulent field isolates clustered within the Neethling-like clade, suggesting continued historical circulation of Neethling-like viruses (69). Unfortunately, these genomes were not publicly available at the time of our analyses. Within the Neethling-like clade, two recent genomes from Serbia and Croatia cluster closely with vaccine genomes and represent cases with adverse reactions to live attenuated vaccines as reported previously (12, 17, 18) and supported by the sampling dates fitting with the implementation of mass vaccination in the Balkan peninsula in 2016.

Removing highly passaged vaccines, vaccine-associated strains, and recombinant viruses from the data set, a spatially explicit reconstruction of the global dissemination history of wild-type LSDV was possible. This placed the origin of LSDV wild-type viruses in sub-Saharan Africa with a timing coherent with the first description of the disease in northern Rhodesia (Zambia) in 1929 and South Africa in 1944 (7). Although the full genome sampling of endemically circulating and spreading LSDV lineages in Africa and the Middle East is extremely patchy, we confirm the endemic circulation of LSDV wild-type in sub-Saharan Africa and its spread from there to the Middle East. For the particular case of endemic LSDV circulation, detailed public whole genome information is missing for most of the historical and recent endemic circulation in Africa and the Middle East, resulting in the high degrees of uncertainty in the geographical locations of ancestral sequences yielded by continuous phylogeographic analyses. Epidemiological data suggest that, from the Middle East, LSDV further spread to Europe as well as to Central Asia (5). Interestingly, the placement in Turkey and Israel of multiple internal nodes of our spatially explicit reconstruction of the LSDV wild-type dispersal history confirms the pivotal role of the Middle East in LSDV global epidemiology and is consistent with the suspected circulation and diversification of LSDV in the Middle East prior to further dissemination to Europe and Asia (5). The haplotype network confirms this pivotal role of the Middle East from which LSDV spread to both Europe and the Caucasus and central Asia (5). Moreover, the placement of Turkey and Israel in the haplotype network is consistent with the findings of risk assessment studies exploring the spatial and temporal risk of LSD, which identified similar regions in the Middle East at high risk of LSDV transmission (77, 78).

Our analyses also confirm the direct link between a commercial vaccine derived from a historical Kenyan strain and recent genomes from India and Bangladesh (32, 33). Interestingly, the viral genome most closely related to the Indian viruses represents a live attenuated vaccine batch produced from a Kenyan isolate, KSGP 0240, from 1974 [KX683219 (15)]. Although separated in sampling time by 45 years (strain used for the KSGP vaccine) and 54 passages in lamb cell culture (lab attenuation history), coding sequences of both viruses differ by only three single nucleotide polymorphisms. In comparison, endemically circulating viruses in Africa between 1999 and 2018 show the accumulation of more than 35 SNPs over a 19-year period. In fact, the genetic similarity between LSDV from the Indian subcontinent and African strains (including the KSGP 0240-based live attenuated vaccine) resembles more the genetic similarity between Neethling strain-based vaccine batches and those occasionally isolated from animals affected by vaccination side effects in Europe. This suggests that anthropogenic involvement in the recent release of KSGP-like strains in East Africa, and/or on the Indian subcontinent is more likely than the continued endemic circulation of wild-type viruses in East Africa followed by introduction to India. However, the limited number of coding differences between the East African and Indian LSDV genomes has resulted in the truncation of open reading frames encoding kelch-like proteins (LSD_19 and LSD-144 genes) in the Indian viruses: a genetic change that is likely to have phenotypic consequences involving virulence and host range (32). A more representative whole genome sampling (from biobank samples) of the endemically circulating LSDV lineages in East Africa between the 1970s and the time when the introduction to India occurred is needed to pinpoint the most probable introduction sources of wild-type LSDV on the Indian subcontinent.

As mentioned above, although the importance of recombination-induced bias in phylogenetic analyses has been repeatedly stressed (28, 29), this issue was not addressed in previous phylogenetic analyses of whole LSDV genomes (e.g. 25, 30, 31 ). We confirm the emergence of at least five different vaccine-like recombinant viruses in livestock in central Asia (24, 25). A single recombinant strain [R4; see reference (24)] further spread to large parts of Asia. An advanced deep sequencing and bioinformatics investigation of vaccine batches used in Kazakhstan consistent with the time of the emergence of these vaccine-like recombinants suggested that these recombinants most likely resulted from mixed strains during vaccine seed production rather than from mixed infection of cattle with wild-type and vaccine strains (24). No direct evidence exists for the spontaneous recombination of capripox viruses in dually infected animals in the field. Removing recombination signals from our maximum likelihood analyses elucidates the vaccine origin of recombinant viruses within the “Neethling vaccines” clade. Specifically, our analysis (compared to analysis lacking correction for recombination) implicates the vaccine strains as the main contributors of genetic material to the five recombinant lineages with the remainder of the genomes of these lineages having been derived from parental genomes in the wild-type clade.

Both our assessments of recombination events and temporal signal constitute particularly important analytical steps when attempting to reconstruct the evolutionary and dispersal history of DNA viruses, which have more slowly evolving and larger genomes than RNA viruses and are often subject to recombination. In the context of the limited genetic variability available in slowly evolving DNA virus populations, both the accuracy of the generated genomic data (which can be substantially impacted by a single miscalled SNP) and the suitability of phylodynamic methods have an important impact on the outcomes of the analyses. Presenting a rare instance of detailed whole genome sampling in a time-constrained outbreak of a DNA virus that is well-adapted to its cattle host, we could formally evaluate the geographic scale at which a correlation existed between genetic and geographical distance. Importantly, we also conducted an analysis of recombination breakpoints, which, if not taken into account, can introduce severe biases during phylogenetic reconstruction as evolutionary models assume that the evolutionary histories of the analyzed sequences can be represented by a single phylogeny.

The current global LSDV situation entails the occurrence in the field of wild-type lineages, vaccine lineages, and at least one vaccine-like recombinant strain (R4) that is consistently spreading in Asia. As LSDV genomic variation is low and randomly distributed over the genome and because recombination is also a potentially problematic issue, the generation of high-quality full genome sequences is extremely relevant. However, our analyses also confirm that, as can be expected due to low LSDV evolutionary rates, the geographical resolution of genetic variation within LSDV epidemic outbreaks such as that occurring between 2015 and 2017 in southeastern Europe is too low to trace virus dispersal with either fine-grained geographical (within country) or fine-grained temporal (within year) resolution. We also show that full genome phylogenetic analyses should avoid the impact of recombination in order to accurately reflect the evolutionary history of LSDV. Haplotype networks provide an interesting visualization of all sequence variants circulating in an epidemic providing limited genetic variability. Specifically for the southeastern European outbreak investigated in the present study, we observed that although several sequence variants were present at the same time and within countries, these do not cluster by time or country of origin. We formulate the following recommendations for the informative use of LSDV whole genome sequencing: (i) to increase both current sampling and WGS of samples in endemic regions, and the WGS of biobanked historical samples collected during outbreaks or in areas of endemic circulation; (ii) to consistently sample and WGS index cases in new geographical areas, unexpected epidemiological settings, or following instances of disease reoccurrences to maximize chances of detecting recombinants or unexpected long-distance LSDV movements (e.g., eastern Africa to India/Bangladesh); and (iii) to carefully assess the occurrence of recombination events and to evaluate the temporal signal within the data set prior to conducting time-scaled phylogeographic reconstructions. Following these recommendations, and preferentially using standardized methodologies in an international collaborative effort, properly targeted whole genome sequencing of capripox viruses can provide important insights to better understand viral evolutionary and dispersal histories: insights that would be useful for the assessment and optimization of control strategies.

## Data Availability

NCBI GenBank accession numbers and linked metadata of the LSDV genomes sequenced in the present study are shared in Table S1. R scripts and related files needed to run all the population genetic, phylogenetic, and phylogeographic analyses (including BEAST XML files) are all available at https://github.com/sdellicour/lsvd_analyses.

## SUPPLEMENTAL MATERIAL

Table S1 jvi.01394-23-s0001.pdf GenBank accession numbers and metadata of the LSDV genomes sequenced in the present study (blue) and public data used as reference genomes.

ASM does not own the copyrights to Supplemental Material that may be linked to, or accessed through, an article. The authors have granted ASM a non-exclusive, world-wide license to publish the Supplemental Material files. Please contact the corresponding author directly for reuse.
