# Supplementary material for: Complete genome reconstruction of the global and European regional dispersal history of the lumpy skin disease virus
Source: J Virol. 2023 Oct 31;97(11):e01394-23. doi: 10.1128/jvi.01394-23 (PMC10688313; doi:10.1128/jvi.01394-23)
Supplement: Table S1 — GenBank accession numbers and metadata of the LSDV genomes sequenced in the present study (blue) and public data used as reference genomes. [file jvi.01394-23-s0001.pdf]

Table S1. GenBank accession numbers and metadata of the LSDV genomes sequenced in the present study (blue) and public data used as reference genomes. Additional metadata kindly provided as personal communication by * Dr. Fahriye Saraç (Pendik Veterinary Control Institute) and ** Dr. Umberto Molini (Central Veterinary Laboratory, Windhoek, Namibia). The latitude and longitude of the sampling location were estimated using Google Maps (https://www.google.com/maps/; accessed on 15/03/2023) considering the center of the most precise administrative unit provided in the metadata.

GenBank accession	strain name	sampling date (dd/mm/yyy)	country	location details	geographical precision	latitude, longitude	Host species
**OR134837**	LSDV/Albania/790/2017	12/06/2017	Albania	Devoll > Poger > Bicke	municipality	40.665262633518005, 20.897221910129662	cattle
**OR134832**	LSDV/Albania/1000/2017	8/08/2017	Albania	Skrapar > Potom > Qafe	municipality	40.48740325877378, 20.30138524995288	cattle
**OR134833**	LSDV/Albania/1573/2016	16/08/2016	Albania	Has > Fajza > Branoge	municipality	42.158472468508435, 20.316930449294585	cattle
**OR134834**	LSDV/Albania/1707/2016	19/08/2016	Albania	Kukes > Shishtavec > Kollovoz	municipality	41.994098179669976, 20.548877630433957	cattle
**OR134835**	LSDV/Albania/4192/2016	25/11/2016	Albania	Kavaje > Gose > Ballaj-Vile	municipality	41.05332003006695, 19.551308042599743	cattle
**OR134836**	LSDV/Albania/4770/2016	14/12/2016	Albania	Elbasan > Librazhd > Pishkash	municipality	41.09703662843592, 20.511042781491245	cattle
KY829023	Evros/GR/15	18/08/2015	Greece	East Macedonia > Evros	municipality	41.24627930183899, 26.135634484697338	cattle
**OR134838**	LSDV/Greece/314/2016	14/07/2016	Greece	Peloponesos > Patra-Ahaia	municipality	38.23574659272103, 21.742206199234325	cattle
**OR134839**	LSDV/Greece/386-16/2016	25/08/2016	Greece	Thessaly > Larisa	municipality	39.63153227442676, 22.413845774212003	cattle
**OR134840**	LSDV/Greece/478/2016	7/11/2016	Greece	East Macedonia > Evros	municipality	41.24627930183899, 26.135634484697338	cattle
**OR134841**	LSDV/Greece/498A/2016	25/11/2016	Greece	Epirus > Preveza	municipality	38.96118045624831, 20.748389862646906	cattle
**OR134842**	LSDV/Greece/715/2015	20/11/2015	Greece	Central Macedonia > Halikidiki	municipality	40.41817707169461, 23.53508083134553	cattle
**OR134843**	LSDV/North_Macedonia/5000/2016	04/2016	North Macedonia	Demir Kapija	municipality	41.40993426051095, 22.24214061599857	cattle

**OR134844**	LSDV/North_Macedonia/5011/2016	05/2016	North Macedonia	Berovo	municipality	41.70758639927124, 22.848257883707024	cattle
**OR134845**	LSDV/Serbia/4592/2016	7/06/2016	Serbia	Bujanovac > Ljiljance	municipality	42.43269597209552, 21.81168009882402	cattle
**OR134846**	LDSV/Serbia/5887/2016	19/07/2016	Serbia	Trstenik > Rajinac	municipality	43.70971847172978, 20.983928983914613	cattle
**OR134847**	LSDV/Serbia/6040/2016	22/07/2016	Serbia	Nova Varoš > Bukovik	municipality	43.420588476346516, 19.95627802387173	cattle
**OR134849**	LSDV/Serbia/7695/2016	24/09/2016	Serbia	Novi Pazar > Netvrdje	municipality	43.11833150920528, 20.56055891095145	cattle
**OR134848**	LSDV/Serbia/6402/2016	30/07/2016	Serbia	Žitorađa > Toponica	municipality	43.14867834210243, 21.687579284871344	cattle (vaccine)
MT643825.1	210LSD-249/BUL/16	15/06/2016	Bulgaria	Yambol region	province	42.48110049877691, 26.494585755855567	cattle
MN642592.1	Kubash/KAZ/16	7/07/2016	Kazachstan	Atyrau region	admin region	47.101730512065025, 51.95277182579847	cattle
KX894508.1	LSDV/ISR/155920/2012	19/12/2012	Israel		country	31.0296395946432, 34.573220816273924	cattle
MT992618.1	KZ-Kostanay-2018	09/2018	Kazachstan	Kostanay	coordinates	53.11995490820831, 63.380064370831406	cattle
MN995838.1	Turkey/pendik/2014 *	13/08/2014	Turkey	Central Anatolia > Tokat province	admin region	40.333646380225844, 36.51514383395085	cattle
MH893760.2	LSDV/Russia/Dagestan/2015	2015	Russia	Dagestan	province	42.58355248639863, 47.02114552639802	cattle
ON400507.1	208/PVNRTVU/2020	2020	India	Telangana	municipality	17.894558879255793, 79.13439929348955	cattle
OM984485.1	LSDV XJ201901	2019	China	Xinjiang	autonomous region	40.27248910572992, 85.43808568834291	cattle
OM793603.1	LSDV_Russia_Khabarovsk_2020	2020	Russia	Khabarovsk	municipality	48.470445975122864, 135.12640709966982	cattle
OM793602.1	LSDV_Russia_Tomsk_2020	2020	Russia	Tomsk	municipality	56.51253326994625, 84.99828066741976	cattle
OL542833.1	LSDV/Russia/Tyumen/2019	2019	Russia	Tyumen	municipality	57.20190571754525, 65.50692149522656	cattle
ON152411.1	LSDV72/PrachuapKhiriKhan/Thailand/2021	21/09/2021	Thailand	Prachuap Khiri Khan	admin region	11.80234266125317, 99.78879106545452	cattle
OM530217.1	LSDV/Russia/Saratov/2019	2019	Russia	Saratov	municipality	51.5509402202156, 45.97469921287834	cattle
OK318001.1	LSDV-V/281-Nigeria	05/2018	Nigeria	Plateau State > Vom	municipality	9.730311532157396, 8.791256890522522	cattle

MW699032.1	LSDV/Russia/Dagestan/2015_75_passage	2015	Russia	Dagestan	province	42.58355248639863, 47.02114552639802	experimental (75 passages)
MT007950.1	Namibia_2016_9F **	3/10/2016	Namibia	Otjozondjupa	coordinates	-20.007159, 18.529314	cattle
ON616408.1	LSDV/NMG/2020	2020	China	Inner Mongolia	autonomous region	42.928711827512174, 113.26954784087641	cattle
OP688129.1	LSDV/Bangladesh/V395.1/2021	2021	Bangladesh	Mymensingh distric	admin region	24.793509365875533, 90.41898480172519	cattle
OP688128.1	LSDV/Bangladesh/V392.1/2021	2021	Bangladesh	Mymensingh distric	admin region	24.793509365875533, 90.41898480172519	cattle
OP297402.1	LSDV-WB/IND/19	2019	India		country	22.946892964646445, 79.52002448908648	cattle
OP508345.1	China/Xinjiang/Cattle/Aug-2019	1/08/2019	China	Xinjiang	autonomous region	40.27248910572992, 85.43808568834291	cattle
OM033705.1	LSDV/Thailand/Yasothon/2021	24/04/2021	Thailand	Yasothon	admin region	15.776488637066748, 104.15674409210172	cattle
MZ577073.1	20L42_Quyet-Thang/VNM/20	28/10/2020	Vietnam		coordinates	21.580700, 105.789300	cattle
MZ577074.1	20L43_Ly-Quoc/VNM/20	30/10/2020	Vietnam		coordinates	22.775800, 106.781900	cattle
MZ577075.1	20L70_Dinh-To/VNM/20	17/11/2020	Vietnam		coordinates	21.062600, 106.031500	cattle
MZ577076.1	20L81_Bang-Thanh/VNM/20	29/11/2020	Vietnam		coordinates	22.657000, 105.723200	cattle
MW732649.1	LSDV/HongKong/2020	1/12/2020	Hong Kong		admin region	22.319300, 114.169400	cattle
MW355944.1	China/GD01/2020	07/2020	China	Guangdong	autonomous region	23.131700, 113.266300	cattle
MT134042.1	LSDV/Russia/Udmurtiya/2019	3/03/2019	Russia	Udmurtiya	province	57.067000, 53.027800	cattle
MH646674.1	LSDV/Russia/Saratov/2017	2017	Russia	Saratov	municipality	51.5509402202156, 45.97469921287834	cattle
OL752713.1	LSDV/KM/Taiwan/2020	17/08/2020	Taiwan	Kinmen Island	municipality	24.45818577393893, 118.3978465747639	cattle
OK422492.1	LSDV/Cattle/India/2019/Ranchi-1/P10	31/12/2019	India	Ranchi	municipality	23.354542402820883, 85.31023785650454	cattle (10 p Vero)
MW656253.1	LSDV/280-KZN/RSA/2018	2018	South Africa	Kwazulu Natal	province	-28.666319903323643, 30.75823826180729	cattle
MW656252.1	LSDV/Haden/RSA/1954	1954	South Africa		country	-30.559500, 22.937500	cattle
MN636843.1	LSD-148-GP-RSA-1997	1997	South Africa	Gauteng > Onderstepoort	municipality	-25.640496278383505, 28.173819466310725	cattle
MN636842.1	LSD-220-2-NW-RSA-1993	1993	South Africa	North West > Potchefstroom	municipality	-26.68156557796832, 27.003573406221026	cattle

MN636841.1	LSD-220-1-NW-RSA-1993	1993	South Africa	North West > Potchefstroom	municipality	-26.68156557796832, 27.003573406221026	cattle
MN636840.1	LSD-248-NW-RSA-1993	1993	South Africa	North West > Potchefstroom	municipality	-26.68156557796832, 27.003573406221026	cattle
MN636839.1	LSD-103-GP-RSA-1991	1991	South Africa	Gauteng > Onderstepoort	municipality	-25.640496278383505, 28.173819466310725	cattle
MN636838.1	LSD-58-LP-RSA-1993	1993	South Africa	Limpopo > Polokwane	municipality	-23.902429914540445, 29.446568409014258	cattle
MN636838.1	Kenya/1958	1958	Kenya		country	-0.023600, 37.906200	cattle
MK441838.1	Herbivac LS batch 008 vaccine	2011	South Africa		country	-30.559500, 22.937500	vaccine batch
MG972412.1	Cro2016	2016	Croatia		country	-0.023600, 37.906200	cattle (vaccine)
KX764643.1	SIS-Lumpyvax vaccine	1999	South Africa		country	-30.559500, 22.937500	vaccine batch
KX764645.1	Neethling-LSD vaccine-OBP		South Africa		country	-30.559500, 22.937500	vaccine batch
KX683219.1	KSGP 0240 vaccine	1974	Kenya		country	-0.023600, 37.906200	vaccine batch
AF325528.1	Neethling 2490	1958	Kenya		country	-0.023600, 37.906200	cattle (16 p in lamb testicle cell culture)
AF409138.1	Neethling vaccine LW 1959	1959	South Africa		country	-30.559500, 22.937500	vaccine batch
AF409137.1	Neethling Warmbaths LW	1999	South Africa	Warmbaths	municipality	-24.86801620964314, 28.270353284621134	cattle
OM105589.1	LSDV/China/XJ01/2019	9/08/2019	China	Xinjiang Uygur Autonomous Region	autonomous region	40.43024751915946, 85.6904893955637	cattle
OM803092.1	China/GX01/2020	10/2020	China	Guangxi Zhuang Autonomous	autonomous region	23.814620971471136, 110.2167063970519	cattle
OM803091.1	China/GD02/2020	06/2020	China	Guangdong Province	autonomous region	23.736140034712463, 114.55126225616479	cattle
